# The Design and Evaluation of Online Interactive Learning in an Undergraduate Nutrition Course

**DOI:** 10.3389/fnut.2022.811103

**Published:** 2022-03-15

**Authors:** Katherine M. Livingstone, Catherine M. Milte, Susie Macfarlane, Julie Woods, Alison Booth

**Affiliations:** ^1^Institute for Physical Activity and Nutrition, School of Exercise and Nutrition Sciences, Deakin University, Geelong, VIC, Australia; ^2^Deakin Learning Futures, Deakin University, Geelong, VIC, Australia

**Keywords:** design, evaluation, online, interactive, learning, undergraduate, nutrition

## Abstract

Understanding factors that promote student engagement with online learning environments is important for benchmarking and improving the quality of teaching in a digital era. This study aimed to describe the online interactive content created for delivery of an undergraduate nutrition course and to evaluate student engagement with the online interactive content. We collected online questionnaire data in 2018 and 2019 from two cohorts of students enrolled in a Deakin University undergraduate nutrition unit. Two-sample unpaired *t*-tests were used to examine differences in participant engagement with online topic guides between static text-based and interactive content. A total of 89 participants (19–56 years) were included. Sixty four of students reported always/usually reading static text-based topic guides most weeks and 64% perceived them as moderately/highly effective. While 60% of participants reported reading the online interactive topic guides most weeks and 93% perceived them as moderate/highly effective. Most participants indicated the interactive topic guides were more effective than static text-based topic guides they experienced in other courses (76%). Hours dedicated to the online interactive topic guide were higher (6.4 SD 2.9 vs. 1.7 SD 1.7 h; *P* < 0.001) as was the rating of how engaging the topic guides were (7.2 SD 1.6 vs. 6.7 SD 2.5; *P* = 0.008). These findings suggest that interactive content is more engaging. However, this content may not be accessible to all students, and so familiarization and training prior to engaging in an interactive online unit may be needed.

## Introduction

High quality online learning environments are needed to navigate the emerging challenges and opportunities of a digital era and the advent of COVID-19 (1). However, the characteristics of the online learning environment vary (2), and the quality of the student experience and its impact on engagement are likely to vary between diverse online learning environments and disciplines (3). In addition, online content can vary in its degree of interactivity, ranging from online static text to highly interactive activities, such as multiple-choice questions and engagement with peers (4). High quality interactive online content enables students to receive immediate formative feedback, encourages critical thinking and better supports students to meet their learning outcomes (5, 6).

There is evidence that students are more engaged when curricula use online interactive content (7, 8). Nonetheless, engagement is likely to differ depending on the learning design (9, 10). To date, most research on nutrition student engagement has focused on in-person interactive learning and flipped classroom active learning approaches (11–14). A paucity of studies has examined engagement with online interactive content in nutrition undergraduate students. In a study in 29 US undergraduate students, formative online assessment was an effective and feasible method to improve student engagement (15). In contrast, in a study of 31 UK undergraduate nutrition and dietetics students, students reported that online content was engaging, but they still preferred face-to-face learning (16). In both studies there was limited reporting of how the online content was designed, or what it included, thus the optimal design of engaging online learning material is unclear. While commercial platforms have been developed for increasing online student engagement, these may not be appropriate for all settings. Moreover, the learning theories that underpin the design of online learning for students vary, and the evidence is mixed for the optimum balance between interactivity and cognitive load (17, 18).

Understanding factors that promote student engagement with online learning environments is important for benchmarking and improving the quality of teaching in a digital era (19–21). Further, there is a need to describe the transition from static text-based learning material to online interactive content within the context of learning design theories, such as constructivism and cognitive load theory. Such information will inform the design of effective online learning for nutrition students, which is of relevance in a digital era and in the advert of COVID-19. Thus, this study aimed to describe the online interactive content created for delivery of an undergraduate nutrition course and to evaluate student engagement with the online interactive content.

## Methods

### Online Interactive Topic Guides

The design of the online content was grounded in Charmaz’s constructivist approach to learning theory (22), where past knowledge is considered the base on which new ideas will be built. Constructivism has been adapted for use with the online learning environment (23), and was applied in this study by focusing on learning design to encourage active engagement, or participation, with the online content (4). The learning design of the interactive online content also considered strategies to minimize extraneous cognitive overload when including interactive content, such as segmenting content and being mindful of coordinating visual and verbal cues (21). This approach was based on the cognitive load theory, where well-designed, low to moderate interactivity is likely to improve engagement with minimal increase in extraneous cognitive load (17).

The design of the online interactive content was led by the lead author (KML), who is a Registered Nutritionist with a Graduate Certificate in Learning and Teaching (Higher Education). Content was reviewed and pilot tested with members of the learning design and teaching team, which included the undergraduate Nutrition Science Course Director, the Associate Head of School Teaching and Learning for Food and Nutrition and Deakin University’s learning design team.

**FIGURE 1:**
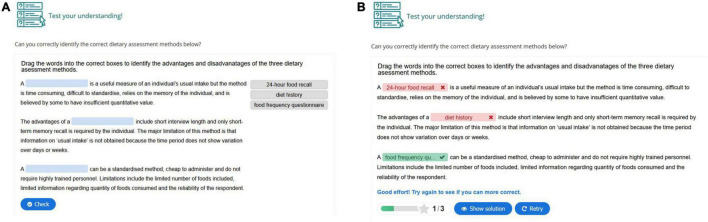
Example of a drag and drop online interactive activity before **(A)** and after **(B)** a student’s attempt to complete the activity. Screenshots © Deakin University. All Rights reserved. Reproduced with permission by Deakin University.

**FIGURE 2:**
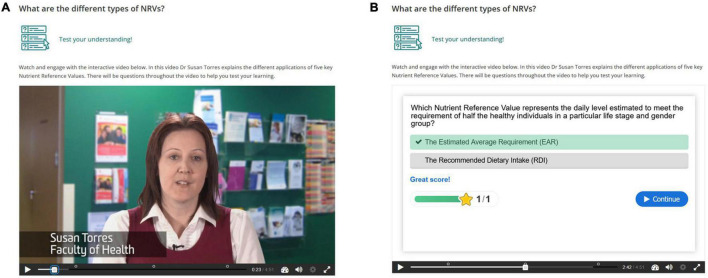
Example of an online interactive video **(A)** with an activity imbedded within the video for the student to complete **(B)** Screenshots © Deakin University. All Rights reserved. Reproduced with permission by Deakin University.

**FIGURE 3:**
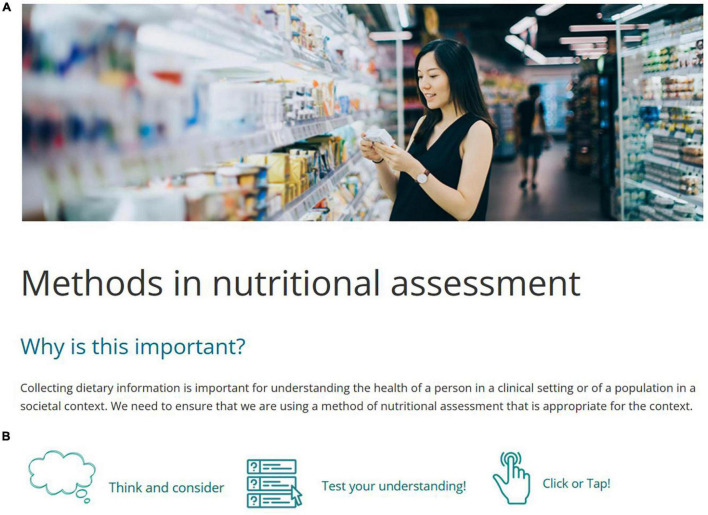
Example of visually rich manner information presented in the online interactive topic guides, with images **(A)** and symbols **(B)** used to signpost sections of content and activities. Screenshots © Deakin University. All Rights reserved. Reproduced with permission by Deakin University. Stock images © Getty Images. Originally reproduced under licence and within this article by permission (https://www.gettyimages.com.au).

Each hypertext mark-up language (HTML) page was designed to include interactive content and formative assessment activities including multiple choice questions, interactive text, images and videos, with opportunities for students to answer questions, solve problems and explore case studies. The authors (KML and SM), with input from the learning design team, created the interactive content using HTML5 Package (HP5) and embedded content into HTML pages. An example of an interactive activity is presented in Figure 1. In this “drag and drop” activity students were presented with a section of text describing three dietary assessment methods with missing words, and were asked to drag the words into the correct boxes to identify the advantages and disadvantages of each method. Immediate feedback was provided with the option to retry until the correct responses were achieved. Interactive explanatory materials were also provided to students in the form of video notes from the lecturer describing a key concept. An example of an interactive video is presented in Figure 2, where students were presented with a video of the lecturer explaining the different applications of five key Nutrient Reference Values, and at certain points were prompted to answer a question to test their understanding of these Nutrient Reference Values before the video continued. Information in the online content was also presented in a more visually rich manner than the PDF documents, with images and symbols used to signpost sections of content and activities (Figure 3). A comment from the lecturer was also placed on each HTML page to provide a sense of teacher presence. For example, “Now that we know more about methods for collected dietary information, we will take a look at how dietary information is collected at a national level in the next section.”

### Evaluation

To evaluate student engagement with the online interactive content, we collected data between June and October in 2018 and 2019 from two cohorts of students enrolled in a Deakin University undergraduate nutrition unit (Lifespan Nutrition). Lifespan Nutrition is a core unit of the undergraduate Bachelor of Nutrition Sciences course offered by the School of Exercise and Nutrition Sciences. A combined total of 576 students were enrolled in the 2018 and 2019 units. The unit is delivered across Victoria, Australia, as a hybrid in-person/online campus as well as online for students enrolled online-only. Students enrolled in a hybrid in-person/online campus attend weekly 2-h lectures and seminar discussions in-person or online. Online-only students join these lectures *via* livestream and attend online synchronous seminars. Students are able to interact with peers and educators during in-person lectures and seminars or *via* written chat if joining *via* the livestreaming. Deakin University’s Learning Management System Blackboard Ultra also provides a Discussion Board monitored by educators on which all students can post and respond to questions at any time. All students had access to weekly topic guides. In the 2018 unit, students had access to online static text-based topic guides only. These topic guides were in the form of a structured PDF document and provide content that is more detailed than that presented *via* oral presentation with PowerPoint slides in the face-to-face lectures. In the 2019 unit, students had access to online interactive topic guides in the form of HTML pages instead of static text-based topic guides. The content and sequence of information in these topic guides were the same in both units, but the modality and format in which the information was presented was different.

### Study Measures

Consenting students were asked to complete an anonymous online questionnaire delivered using Qualtrics online survey software. The online survey collected information on demographic characteristics, the enrolled course, and opinions on the effectiveness of the unit resources for meeting learning outcomes and how engaging the resources were (Supplementary Table 1). The Deakin University Ethics Committee approved this study (HEAG-H 151_2018). Demographic data collected included age (categorized into 18–21 years and 22–56 years), sex (female or male) and campus (categorized into hybrid in-person/online, or online-only). Information on personal learning experiences within the unit were collected. Participants were asked how often they read the topic guide, with response options of always, usually, sometimes, rarely, never. Participants reported how effective they found the topic guides for meeting the unit learning outcomes (scale 0–10). These were re-grouped into tertiles for descriptive purposes: low, moderate and high effectiveness. Information was collected on how many hours per week participants studied the topic guides and how engaging they were (scale 0–10). In the 2019 unit with interactive online topic guides, participants were asked if they preferred the online interactive topic guide to the static text-based topic guide (Yes, No). A short description of static text-based and online interactive topic guides was included in the questionnaire for standardization; an interactive topic guide was described as including online activities and opportunities to test learning and get immediate feedback/answers.

### Data Analysis

Two-sample unpaired *t*-tests were used to examine differences in participant engagement with topic guides between static text-based and interactive topic guides using Stata version 15.0 (StataCorp). *P* < 0.05 was considered statistically significant. Qualitative information was collected on students’ perceptions of topic guides using an open-ended free-text question asking participants to rate the interactive topic guides used in this unit when compared to a text-based topic guide and to then explain why (Supplementary Table 1). Due to this being an optional question, responses were presented in a narrative form, with example quotations.

## Results

A total of 89 participants were included in this evaluation (2018: *n* = 47 and 2019: *n* = 42). The response rate for both surveys was 15%. Participants were aged 19–56 years, were predominantly female and enrolled in the hybrid in-person/online campus in both the 2018 static text-based and 2019 interactive online topic guide units.

In the 2018 unit with online static text-based topic guides, the majority of participants reported always/usually reading static text-based topic guides most weeks (64%) and 46% of participants perceived them as moderately/highly effective. In the 2019 unit with online interactive topic guides, 60% of participants reported reading online interactive topic guides most weeks and almost all participants perceived them as moderate/highly effective (90%). Most participants indicated that the interactive topic guides were more effective than static text-based topic guides they experienced in other courses (76%). Hours dedicated to the online interactive topic guide were significantly higher in the interactive topic guide condition as was the rating of how engaging the topic guides were, compared with the online static text-based topic guide (Table 1).

**TABLE 1:** Participant engagement with online static text-based topic guides and online interactive topic guides.

Characteristic	Percentage or mean (SD)		*p*-value
	Static text-based topic guide (*n* = 47)	Interactive topic guide(*n* = 42)	
Frequency read topic guides (%)			0.70
Always	34.0	40.5	
Most or some weeks	63.8	59.5	
Never	2.1	0.0	
Effectiveness of topic guides (%)			0.45
Low	63.8	9.5	
Moderate	23.4	38.1	
High	12.8	52.4	
Topic guide			
Hours using guide per week (mean, SD)	1.7 (1.7)	6.4 (2.9)	<0.001
Engagement of text-based guides (0–10; mean, SD)	6.7 (2.5)	7.2 (1.6)	0.008
Interactive guides were more effective (%)		76.2	

*P-values are from ANOVAs (categorical variables) and two-sample unpaired t-tests (binary variables).*

In the 2019 unit with online interactive topic guides, participants who perceived the interactive topic guide as more effective in helping them meet learning outcomes described them as:

*“If I get something wrong I investigate to consolidate my lea*rning.” (Participant 33, female, 30 years). *“Interactive topic guides are engaging and keeps you interested as opposed to reading through a entire page of words.”* (Participant 16, female, 22 years). *“Interactive learning activities and opportunities would allow for further testing of knowledge and encourage more time put into the unit. These don’t necessarily have to be graded but even just for the chance to test our own knowledge and allow students to see what areas they need to put more time into.”* (Participant 11, male, 20 years). *“I’m a visual learner”* (Participant 60, female, 21 years).

Participants who perceived the interactive topic guide as less effective in helping them meet learning outcomes described them as:

*“I don’t like having to navigate through various screens to find information I’m looking for.”* (Participant 4, female, 43 years). *“Quite a lot of information, kind of overwhelming.”* (Participant 28, female, 31 years). *“Not everyone learns best online.”* (Participant 21, female, 31 years).

## Discussion

This study aimed to describe and evaluate the delivery of online interactive content in an undergraduate nutrition unit. Our main findings showed interactive content was rated as more engaging, and that students also spent more time with the interactive content than the static content. However, the online interactive content may not be accessible to all students, and so familiarization and training prior to engaging in an interactive online unit may be needed. These experiences with online interactive content, activities and feedback can be used to inform the design of online learning in nutrition curricula, which is timely given the greater need to actively engage students in remote learning as a result of COVID-19.

Our finding of a preference for online interactive learning in undergraduate nutrition studies is consistent with previous research in nutrition and dietetics (16) and other fields (3, 24, 25). In a study of 2,196 students from 29 Austrian universities, students indicated online learning provided a clear and coherent structure and advocated for its use when developing skills in self-regulated learning (25). However, there is evidence that the design of online learning material is imperative for delivering coherent and engaging content. In particular, studies have shown students prefer formative assessment tools, such as practice quizzes and case studies, over text readings and PowerPoint slides (26). Thus, while online interactive content is designed based on best practice learning paradigms, such as constructivism and cognitivism (4), the learning materials are also designed with the aim of improving engagement. For example, the inclusion of interactive and multimedia content enables the student to be an active agent in their learning and to develop critical thinking and problem-solving skills, while also creating a fun and engaging learning experience for the student (8, 27).

Our study adds to the literature on constructivist learning design adapted for online learning. In constructivist theory, there is a focus on learning design that encourages active engagement, or participation, with the content (4, 28). As described by Carwile, “the notion that students actively construct their knowledge fits within the framework of online education because students are separated from their teachers and expected to have the autonomy to regulate their learning” (29). However, the literature in this area is still emerging, and limited research exists in the field of nutrition education. In a recent nutrition and health virtual classroom case study in Portuguese students, the use of a pedagogical strategy for higher education highlighted the benefit of problem-based learning and online interaction (with peers and teachers) for achieving successful learning outcomes (30). This aligns with the present study, where quizzes, case studies and the presence of the educator in the learning content and discussion forums provided a constructivist and student-centered learning environment. Moreover, the consideration of strategies to minimize extraneous cognitive overload, such as being mindful of coordinating visual and verbal cues, align with previous literature on the optimal design of constructivist online learning (17, 21).

Our findings that participants reported greater engagement is consistent with research in nutrition students, where there were gains from active learning, including formative feedback and opportunities for students to test their learning (11, 15, 31). In a study of 29 US undergraduate nutrition students, the majority of students (76%) preferred the online delivery format to face-to-face delivery, (15) which is on par with the 76% of students in this study who rated the interactive topic guides as more effective than static text-based topic guides. Nonetheless, the transition to a fully online unit may create challenges when re-creating classroom discussions and associated skills acquisition (32) and warrants further investigation. This investigation will be particularly important for nutrition students who have experienced new learning design as a result of COVID-19 (1).

The present study had a number of strengths. To minimize respondent bias due to assessment task results, the questionnaire was administered before students received their main assignment grade. As it was not possible to link participant data with assessment task data, future research should extend these results to compare academic performance. Although no validated measures were used, the mixed-methods design enabled collection of quantitative, as well as contextual, information on student engagement. In addition, the study design and questionnaire were designed based on established constructivist and cognitive load theories and in collaboration with the undergraduate Nutrition Science Course Director and the Associate Head of School Teaching and Learning for Food and Nutrition and Deakin University’s learning design team. A number of limitations should be acknowledged. The response rate in the study was low, suggesting that the generalizability of these findings to the wider student cohort may be limited. Future research should consider strategies for increasing the response rate, such as incorporation of the evaluation into the curriculum or assessment tasks. While most participants were female, this is characteristic of the health-related topic of the unit and participant demographics between years were comparable.

The present study showed higher engagement in undergraduate nutrition students provided with online interactive topic guides than those provided static text-based topic guides. These findings support the use of online interactive learning content grounded in constructivist and cognitive theory. However, the small sample of students included in this study means that this content may not be accessible to all students, and so familiarization and training prior to engaging in an interactive online unit may be needed. As these data were collected prior to the COVID-19 pandemic, future research should extend these findings to identify further opportunities for improving nutrition student outcomes in a blended or fully online learning environment.

## Data Availability Statement

The datasets presented in this article are not readily available because data are not publicly available. Requests to access the datasets should be directed to KML, k.livingstone@deakin.edu.au

## Ethics Statement

The studies involving human participants were reviewed and approved by the Deakin University Ethics Committee (HEAG-H 151_2018). The patients/participants provided their written informed consent to participate in this study.

## Author Contributions

KML, CMM, SM, JW, and AB designed the analysis. KML conducted the statistical analysis and drafted the manuscript. All authors provided a critical review of the manuscript and approved the final version of the manuscript.

## Conflict of Interest

The authors declare that the research was conducted in the absence of any commercial or financial relationships that could be construed as a potential conflict of interest.

## Publisher’s Note

All claims expressed in this article are solely those of the authors and do not necessarily represent those of their affiliated organizations, or those of the publisher, the editors and the reviewers. Any product that may be evaluated in this article, or claim that may be made by its manufacturer, is not guaranteed or endorsed by the publisher.
