# Supplementary material for: The Design and Evaluation of Online Interactive Learning in an Undergraduate Nutrition Course
Source: Front Nutr. 2022 Mar 15;9:811103. doi: 10.3389/fnut.2022.811103 (PMC8965005; doi:10.3389/fnut.2022.811103)
Supplement: Supplementary file 1 [file Table_1.docx]

**Supplemental Table 1.** Summary of questions used in this study^1^

| **Questionnaire item** | **Response option** |
| --- | --- |
| **Demographic characteristics** |  |
| What is your age? | Select from 18-100 years |
| I am [please select one response only] | Male; Female; Other; No response |
| Which campus are you enrolled in? | Burwood (Melbourne); Waurn Ponds (Geelong); Warrnambool; Cloud (online); Other (please specify)^2^ |
| **Unit specific information** |  |
| How often do you read the topic guides?  *The topic guides are the Word documents that contain your weekly learning content. These are designed to complement the weekly classes.* | Always, I read each week’s topic guide; Usually; Sometimes; Rarely; Never, I don’t engage with the topic guides |
| How effective have you found the topic guides in helping you meet the learning outcomes? | Rate from 0-10 |
| On average, how many hours per week do you dedicate to the topic guides? | Select from 0-168 hours |
| In terms of how engaging your learning is, please rate the interactive topic guides used in HSN202 when compared to a standard topic guide.  *The online activities for each topic that allow you to test your learning and get immediate feedback/answers are called the interactive topic guides. Similarly, a classic online word/PDF document that contain your weekly learning content without any interactive activities is called a standard [static text-based] topic guide.* | Rate from 0-10 |
| Have you found the interactive topic guides to be more effective in helping you meet the learning outcomes for this unit this trimester when compared to a standard [static text-based] topic guide? | Yes; No |

1, To avoid use of complex language, the *static text-based topic guides* refered to in this study were refered to as *standard topic guides* in the online survey for student participants.

2, Burwood (Melbourne); Waurn Ponds (Geelong) and Warrnambool were categirised as a hybrid in person/online campus
